# Psychosocial outcomes in young adults with childhood traumatic brain injury: A 20‐year follow‐up study

**DOI:** 10.1111/jnp.70042

**Published:** 2026-04-15

**Authors:** Edith Botchway‐Commey, Nicholas P. Ryan, Louise Crowe, Celia Godfrey, Harry Macleod, Stephen Hearps, Vicki A. Anderson, Cathy Catroppa

**Affiliations:** ^1^ Murdoch Children's Research Institute Melbourne Victoria Australia; ^2^ Royal Children's Hospital Melbourne Victoria Australia; ^3^ Department of Paediatrics University of Melbourne Melbourne Victoria Australia; ^4^ Cognitive Neuroscience Unit Deakin University Geelong Victoria Australia; ^5^ Melbourne School of Psychological Sciences University of Melbourne Melbourne Victoria Australia

**Keywords:** employment, psychosocial outcomes, quality of life, relationship, substance use, traumatic brain injury, young adulthood

## Abstract

We assessed the proportion of childhood TBI survivors who experienced favourable or less favourable psychosocial outcomes (defined as less favourable when 20% or more of the sample report difficulties or unmet needs) compared to controls and to explore factors associated with these outcomes at 20 years post‐TBI. Participants included 54 young adults (age 22–34 years) who sustained mild (*n* = 14), moderate (*n* = 27), or severe (*n* = 13) TBI during childhood, and age and sex‐matched 13 typically developing controls. Outcomes assessed: employment, quality of life, relationships, mental health, offending behaviours, and substance use outcomes. Study‐designed and validated questionnaires (e.g., World Health Organization Quality of Life Bref) were used. Analyses involved descriptive statistics and generalised linear models. A high proportion of TBI participants reported favourable outcomes for offending behaviours (90.7%–94.3%). However, a substantial proportion (24%–87%) of participants reported less favourable outcomes in education, employment, overall QoL, general health, relationships, anxiety, tobacco use, and alcohol use. The TBI group reported significantly lower Overall QoL compared to controls, and the mild TBI group was at a greater risk of reporting less favourable outcomes. Other significant predictors of outcomes were younger age at injury and lower SES at the time of injury. These findings highlight that some psychosocial domains (e.g., relationships) are more affected than others (e.g., offending behaviours) in young adults with childhood TBI. Further research is needed to identify factors influencing psychosocial outcomes and to develop targeted interventions for young adults with childhood TBI.

## INTRODUCTION

Childhood traumatic brain injury (TBI) is one of the leading causes of death and disability globally (World Health Organization, 2006). The developing brain is particularly vulnerable to the effects of TBI, making these injuries especially concerning. TBI during these critical developmental stages can have lasting negative impacts on cognitive abilities, learning, language acquisition, social development, and overall psychosocial functioning (Anderson et al., 2011; Beauchamp & Anderson, 2010; Catroppa & Anderson, 2005; Crowe et al., 2012, 2014; Ryan, 2017). Less favourable long‐term outcomes have been reported in several psychosocial domains, including education, behaviour (including offending behaviours), and quality of life (QoL). However, there is a notable lack of rigorous research examining these outcomes beyond adolescence (Lopez et al., 2022; McKinlay et al., 2014; Ryan, Reyes, et al., 2019), leaving key questions unanswered regarding how young adults who sustained TBI in childhood navigate their everyday lives. Long‐term follow‐up of childhood TBI outcomes is crucial to fully understand the enduring impact of these injuries, identify individuals at higher risk of adverse outcomes, and inform the targeted allocation of healthcare and support resources (Jonsson et al., 2004).

Individuals who sustain TBI in childhood often experience less favourable psychosocial outcomes across critical domains, including education (Anderson et al., 2009; Catroppa et al., 2009; Crowe et al., 2012; Durber et al., 2017; Ilie et al., 2020), employment (Anderson et al., 2009; De Netto & McKinlay, 2020; Sariaslan et al., 2016), QoL (Di Battista et al., 2012; Greenham et al., 2020; Tuerk et al., 2020; Ulgen Tekerek et al., 2023; Von Steinbuechel et al., 2023), mental health (Albicini & McKinlay, 2015; Durish et al., 2018; Sabir et al., 2023), and offending behaviours (Carr, Brandt, et al., 2024; Carr, Hall, et al., 2024), with the greatest impact typically seen in those with severe TBI. A population‐wide Scottish study of individuals who sustained childhood TBI (mild, moderate, and severe) reported poorer educational outcomes in the TBI group (*n* = 4788), particularly among those who had been hospitalised, compared to their peers (*n* = 761,456) 9 years post‐injury (Visnick et al., 2023). Ryan, Noone, et al. (2019) found a significant reduction in health‐related QoL (HRQoL) in TBI compared to controls at 15 years post‐injury, which was associated with severe depression symptoms and social communication difficulties. Reduced emotional and social functioning, and poor peer relationships have also been reported at 4 years post‐injury, even after mild TBI, which were associated with lower socioeconomic status (SES), and poorer parent mental health (Jones et al., 2021). Offending behaviour (e.g., aggression and violence) is also reported in some childhood TBI studies, and has been associated with severe TBI, emotional dysregulation, younger age at injury, and substance use (e.g., alcohol use) (Ryff, 1995).

While previous studies have greatly contributed to our understanding of psychosocial outcomes following childhood TBI, they have provided limited insights into outcomes into young adulthood. As childhood and adolescence bring many transitions and increasing demands for independence and responsibility, definitive conclusions about the long‐term impact of childhood TBI can only be drawn once the individual reaches adulthood. Follow‐up into adulthood also provides an opportunity to assess whether an individual has successfully developed the cognitive and behavioural skills necessary for navigating everyday life (Jonsson et al., 2004). Given the impact of TBI on the developing brain, it is likely that some complex cognitive and behavioural skills essential for this stage may be compromised. A comprehensive evaluation of psychosocial functioning is necessary to identify factors that predict poor outcomes, vulnerable subgroups, and characterize the long‐term support needs for this high‐risk population. This study adopts a holistic definition of psychosocial outcomes, drawing on Ryff's (1995) framework of this concept, which extends beyond the mere absence of distress to include positive self‐esteem, autonomy, fulfilling relationships, purpose, and personal growth. We will examine the following psychosocial domains: education, employment, QoL, relationships, mental health, offending behaviours, and substance use.

The current study has three aims: (1) to determine the proportion of young adults at risk of both favourable and less favourable psychosocial outcomes 20 years post‐childhood TBI compared to a typically developing control (TDC) group; (2) to compare rates of favourable and less favourable outcomes across mild, moderate, and severe TBI groups, and (3) to identify the factors associated with these outcomes in the TBI groups, including *demographic/injury‐related/personal characteristics* (TBI severity, sex, age at injury, SES at baseline/at the time of injury (T1), family functioning at T1, and therapies received up to 15 years post‐injury) and the *child's functional outcomes* (adaptive behaviour and IQ at baseline, and executive function and social skills at 10 years post‐injury). The disparity in timepoints for these potential related factors enables exploration of the influence of early and later developmental factors on outcomes in young adulthood.

Based on findings from the limited available studies (Anderson et al., 2009, 2010; Bellesi et al., 2019; Carr, Brandt, et al., 2024; Sariaslan et al., 2016), it was expected that: (1) compared to the TDC group, young adults with childhood TBI would report less favourable outcomes across most psychosocial domains; (2) a dose–response relationship will be detected, such that individuals with more severe TBI would be more likely to report poorer outcomes those with mild TBI, and (3) psychosocial outcomes will be significantly associated with demographic/personal factors (e.g., TBI severity, sex, age at injury, SES) and functional outcomes (e.g., adaptive behaviour and IQ).

## METHODS

### Design and ethics

This study is part of a larger prospective, longitudinal project examining the long‐term outcomes of childhood traumatic brain injury (TBI) (Catroppa & Anderson, 2005). In this paper, we specifically focus on psychosocial outcomes assessed 20 years post‐injury. This study has ethics approval (HREC Ref No: 30064).

### Participants

The study included 54 young adults who sustained TBI in childhood (age: *M* = 27.7, SD = 3.3), recruited at time of injury (T1) from consecutive admissions to the neurosurgical ward of a Children's Hospital in Australia, between June 1993 and June 1997 (Anderson et al., 2000). Participants were classified as having a mild (*n* = 14), moderate (*n* = 27), or severe TBI (*n* = 13). Inclusion criteria were (Anderson et al., 2000): (1) age at injury between 0 and 12 years; (2) documented evidence of TBI, including a period of altered consciousness at the time of injury; and (3) English speaking. Exclusion criteria were: (1) penetrating head injury; (2) head injury resulting from abuse; (3) history of previous head injury; and (4) any evidence of pre‐existing physical, neurological, psychiatric or developmental disorder. Injury severity classification was based on the following: *Mild TBI* (*n* = 44): Glasgow Coma Scale (GCS) score on admission of 13–15, post‐traumatic amnesia (PTA) <24 h, and no abnormality on CT or MRI scan; *Moderate TBI* (*n* = 81): GCS on admission = 9–12, PTA 1–7 days, and/or abnormalities on CT or MRI scan; and *Severe TBI* (*n* = 47): GCS = 3–8 at the time of admission, PTA >7 days, and abnormalities on CT or MRI scan.

The original study included 172 children with TBI and 35 typically developing control (TDC) participants. Tables S1 and S2 present demographic comparisons of participants and non‐participants (i.e., those who did not participate in this 20‐year follow‐up), and reasons for loss to follow‐up.

### Procedure

All participants provided written consent and completed questionnaires in hardcopies (sent via post) or online via REDCap. A detailed account of the study procedure has been previously published (Botchway et al., 2019, 2020).

### Measures

#### Primary outcomes: Psychosocial functioning at 20 years post‐injury

Six main areas of psychosocial functioning were assessed (education, employment, QoL, relationships, mental health, offending behaviours, and substance use), *generating 16 outcomes/domains*. As indicated in Table 1, scores for each outcome were dichotomised into favourable versus less favourable. Currently, there is no standard definition for ‘high prevalence’ of outcomes in either childhood or adult TBI research. We therefore adopted a ≥20% threshold for less favourable outcomes, drawing on a recent pivotal study in obesity research that classified rates ≥20% as ‘very high’ prevalence (Lobstein & Jewell, 2022). Based on this criterion, outcome domains (e.g., education) were classified as ‘predominantly favourable outcomes’ when ≥80% of participants reported positive outcomes and ‘predominantly less favourable outcomes’ when ≥20% of participants reported difficulties or unmet needs.

**TABLE 1:** Psychosocial outcome measures.

Domain	Item/questionnaire used	Dichotomized outcomes reported	
		Favourable (1)	Less favourable (2)
Education	Which of the following best describes the highest level of education you have currently completed?	Undergraduate, technical, or postgraduate education	High school or lower (i.e., Year 12 or lower)
Employment	What is your current employment status?^a^	Employed	Unemployed
	How satisfied are you about your ability to perform your duties in your current job?^a^	Satisfied	Dissatisfied
QoL	How would you rate your quality of life? *WHOQoL item*	Good overall QoL	Poor overall QoL
	How satisfied are you with your health*? WHOQoL item*	Satisfied	Dissatisfied
Relationships	Which of the following best describes your current relationship status?^a^	In a relationship	Not in a relationship
	How satisfied are you with your personal relationships? *WHOQoL item*	Satisfied	Dissatisfied
	To what extent do you feel your life to be meaningful? *WHOQoL item*	Greatly or extremely meaningful	Minimal or small extent
Mental health	DASS—Depression subscale	Below clinical range	Mild to extremely severe depression
	DASS—Anxiety subscale	Below clinical range	Mild to extremely severe anxiety
Offending behaviours	During the last 12 months how often have you been involved in any of the following situations?^a^ Got involved in a fight. Taken something from a shop without paying. Got in trouble with the law or police?	Never	One or more times
Substance use	How frequently have you used any of the following substances?^a^ Alcohol Cigarettes or tobacco Marijuana or hashish (cannabis)	Less often used (never/once/twice a month/few times a year)	Often used (at least once a week/almost every day)

*Note*: Favourable versus less favourable outcomes were categorized as follows: *education* (Undergraduate/technical/postgraduate vs. Highschool/Lower), *employment status* (employed vs. unemployed), *satisfaction with ability to work* (satisfied vs. dissatisfied), *Overall QoL* (Good vs. Poor), *Overall general health* (satisfied vs. dissatisfied), *relationships status* (in a relationship vs. not in a relationship), *satisfaction with personal relationships* (satisfied vs. dissatisfied), *life is meaningful* (Great/extremely meaningful vs. not/small/moderately meaningful), *depression/anxiety* (normal/mild vs. moderate to extremely severe), *offending behaviours* (Never vs. One or more times), *substance use* (Less often used—never/once/twice a month/Few times a year; Often used—At least once a week/almost every day). Outcome areas (e.g., education) were classified as having ‘predominantly favourable outcomes’ when ≥80% of participants have positive outcomes and ‘predominantly less favourable outcomes’ when ≥20% of participants reported difficulties or unmet needs.

Abbreviations: DASS, Depression, Anxiety, Stress Scale; QoL, quality of life; WHOQoL‐Bref, World Health Organization Quality of Life Bref.

^a^ Data collected using study‐designed questions.

At 20 years post‐injury, items from a study‐designed questionnaire were used to evaluate some outcomes (e.g., education and employment). The Overall QoL and general health items from the World Health Organization Quality of Life‐BREF (WHOQoL‐BREF) were used to assess QoL (Skevington et al., 2004), each scored on a 5‐point scale (1 to 5). Overall QoL ratings ranged from (1) Very Poor, (2) Poor, (3) Neither Poor nor Good, (4) Good, and (5) Very Good. General Health ratings ranged from (1) Very Dissatisfied, (2) Fairly Dissatisfied, (3) Neither Dissatisfied nor Satisfied, (4) Satisfied, and (5) Very Satisfied. For this study, we adopted a conservative approach to defining ‘good’ QoL, to ensure that only clear endorsements of good QoL were categorised as such to better identify individuals needing support. Since individuals selecting option 3 (Neither Dissatisfied nor Satisfied) are more likely be to neither experiencing notable distress or well‐being, their response aligns more with lower QoL than with positive QoL. Hence, scores between 1 and 3 on both items were classified as Less Favourable (i.e., poor outcomes) and those from 4 to 5 Favourable (i.e., good outcomes).

Total anxiety and depression scores of the Depression Anxiety Stress Scale (DASS: 42‐items) were used to assess mental health (Brown et al., 1997; Osman et al., 2012). These scales have 14 items, scored on a 4‐point scale (0 = did not apply to 3 = very much applies). Based on normative cut‐off scores (Brown et al., 1997; Klonoff et al., 1977; Osman et al., 2012), scores within the ‘normal’ range were classified as favourable, whereas scores in the mild to extremely severe ranges were labelled less favourable.

#### Predictors/risk factors

##### Demographic/personal characteristics

Participants' age at injury and injury severity were extracted from RCH medical records at baseline (T1). A study‐designed demographic questionnaire was used to collect data on participants' age and sex at 20 years post‐injury (T6), and the number of therapies received by 15 years post‐injury (T5). The following therapies were evaluated: psychologist, cognitive remediation, physiotherapy, speech therapy, educational support, occupational therapy, social work/case management, and other interventions. SES was assessed at baseline (T1) using Daniel's Scale of Occupational Prestige (Daniel, 1983) which ranks parent occupation on a 7‐point scale of 1.0–7.0, with higher scores representing lower SES. Scores can be categorised into high (<4) or low (≥4) SES. Family functioning (Intimacy) was assessed at T1 using the Family Functioning Scales (FFS), which measures parenting style, with a higher score reflecting more of a given characteristic. The Intimacy subscale was chosen for this paper since it highly correlates with functional outcomes (Noller et al., 1992).

##### Child's functional outcomes

Adaptive Functioning, measured at baseline (T1), was parent‐rated using the Vineland Adaptive Behaviour Scale (Brown et al., 1997), yielding an Adaptive Behaviour Composite (ABC) score (Mean [*M*] = 100, Standard Deviation [SD] = 15), with higher scores reflecting better adaptive functioning. Intellectual Ability at T1 was assessed with the Wechsler Intelligence Scale for Children—3rd Edition (WISC‐3) (Klonoff et al., 1977), and the Full‐Scale IQ (FSIQ: *M* = 100, SD = 15) was used in subsequent analyses. Executive Functioning was rated by parents at 10 years post‐injury (T4) using the Behaviour Rating Inventory of Executive Function (BRIEF) (Daniel, 1983), where higher scores indicate poorer executive function, and scores above 65 reflect clinically significant impairment (*M* = 50, SD = 10). Social Skills were assessed at T4 using the Behaviour Assessment System for Children‐2 (BASC‐2) (Noller et al., 1992), with lower scores (<40) representing poorer social skills (*M* = 50, SD = 10).

### Statistical analysis

Analyses were performed using IBM SPSS Statistics (Version 29). Demographic and functional outcomes were examined using descriptive statistics, and group differences were evaluated using One‐Way Analysis of Variance (ANOVA), *χ* ^2^ tests, Kruskal–Wallis tests, and Mann–Whitney *U* tests. We compared the whole TBI and TDC group, as well as the TBI severity groups.

Aim 1 involved *χ* ^2^ tests comparing differences in the proportion of TBI and TDC participants reporting favourable versus less favourable outcomes across psychosocial domains (education, employment, QoL, relationships, offending behaviours, and substance use). For Aim 2, *χ* ^2^ tests compared rates of favourable versus less favourable outcomes across TBI severity groups. For both aims 1 and 2, outcome domains (e.g., education) were classified as ‘predominantly favourable outcomes’ when ≥80% of participants reported positive outcomes and ‘predominantly less favourable outcomes’ when ≥20% reported difficulties or unmet needs. Aim 3 also focused on the TBI group, and since most outcome variables were not normally distributed, Generalised Linear Models (Binary logistic models) were used to determine whether these factors were significantly associated with each psychosocial outcome domain: (i) *Demographic/personal factors*: TBI severity, sex, age at injury, SES (T1), FFS (T1), self‐reported therapy completed by T5, and other‐reported therapy completed by T5 and (ii) *Functional outcomes*: FSIQ (T1), VABS (T1), BRIEF (T4), and BASC‐2 (T4). Where significant relationships emerged, adjusted models were run, controlling for demographic/personal factors that differed across the groups. Alpha was set to .05 for all other analyses, and confidence intervals are reported, where applicable.

## RESULTS

### Sample characteristics

Demographic comparisons between the original cohort and those who participated in the 20‐year follow‐up are presented in Table S1. Non‐participants were more likely to be male (*χ* ^2^[1, *n* = 172] = 9.33, *p* = .002), had higher SES at the time of injury (median = 4.30, *U* = 2541.50, *p* = .032), and had lower IQ (*p* < .001) than participants.

Characteristics of the 20‐year sample are presented in Table 2, comparing TBI participants who completed the current follow‐up (*n* = 54) with the TDC group (*n* = 13). The two groups differed significantly on IQ (higher in the TDC group, *p* = .003) and proxy‐reported number of therapies (higher in the TBI group, *p* = .029).

**TABLE 2:** Sample characteristics.

	TBI, *n* = 54	TDC, *n* = 13	*p*	Mild TBI, *n* = 14	Moderate TBI, *n* = 27	Severe TBI, *n* = 13	*p*	Significant contrasts
**Demographic/personal characteristics**								
Sex (T6, male) *n* (*%*)	27 (50.0)	8 (61.5)	.455^b^	9 (64.3)	13 (48.1)	5 (38.5)	.392^b^	‐
Age at injury (T1, years), *M* (SD)	6.5 (3.2)	‐	‐	8.4 (3.0)	5.7 (3.0)	6.0 (3.3)	.**035** ^a^	**1 > 2**
Age at follow‐up (T6, years), *M* (SD)	27.7 (3.2)	26.0 (2.1)	.074	29.1 (3.0)	27.1 (3.1)	27.2 (3.4)	.144	‐
SES (T1), *M* (SD)	4.1 (1.1)	3.4 (1.2)	.075	3.7 (1.0)	4.2 (1.1)	4.2 (1.3)	.347	‐
FFS (T1), *M* (SD)	65.3 (8.7)	67.0 (3.9)	.535	62.5 (14.6)	66.0 (5.5)	66.9 (4.7)	.919	‐
Number of therapies (T5—self), *M* (SD)	1.6 (1.8)	.9 (1.0)	.222	.6 (.7)	1.3 (1.5)	3.5 (2.1)	**<.001**	**3 > 1 & 2**
Number of therapies (T5—other), *M* (SD)	1.9 (2.1)	.5 (.7)	.**029**	.3 (.5)	1.8 (1.9)	3.8 (2.1)	.**001**	**3 > 1 & 2**
**Functional outcomes**								
ABC (T1), *M* (SD)	111.3 (16.9)	118.1 (15.8)	.202	112.1 (19.8)	112.6 (15.0)	108.1 (18.3)	.751	‐
FSIQ (T1), *M* (SD)	102.8 (16.2)	118.5 (17.1)	.**003**	105.1 (11.7)	105.7 (15.6)	94.8 (19.6)	.116	‐
BRIEF (T4), *M* (SD)	51.6 (17.8)	43.3 (15.6)	.178	58.7 (15.5)	49.5 (21.2)	57.0 (12.0)	.560	‐
BASC (T4), *M* (SD)	46.0 (21.1)	56.6 (9.2)	.106	54.3 (17.8)	40.6 (25.2)	52.4 (10.7)	.502	‐

*Note*: Analysis are based on One‐Way ANOVA tests, unless otherwise specified. Bold values represent statistically significant differences (*p* < .05).

Abbreviations: ABC, adaptive behavior composite; BASC, behavior assessment system for children; BRIEF, behavior rating inventory of executive function; FFS, family functioning scale; FSIQ, full scale intelligence quotient; SES, Socio‐economic status; TBI, traumatic brain injury; TDC group, typically developing control group. Missing: SES = 8; FFS = 17; Number of therapies (T5—self) = 18; Number of therapies (T5—other) = 19; ABC = 8; FSIQ = 2; BRIEF = 28; BASC = 29.

^a^ Kruskal–Wallis tests.

^b^ Chi‐squared tests. T1 = baseline; T4 = 10 years post‐injury; T5 = 15 years post‐injury; T6 = 20‐years post‐injury. Number of therapies (self/other) was based on how many of these 8 options were endorsed: psychologist, cognitive remediation, physiotherapy, speech therapy, educational support, occupational therapy, social work/case management, and other interventions.

When examining differences across the TBI severity groups, no significant differences were identified on *functional outcomes* (IQ, executive functioning, adaptive behaviour, social outcomes). However, they differed significantly on age at injury (*p* = .035), with the mild TBI group being significantly older than the moderate TBI group (*p* = .032). Group differences also emerged for average number of therapies received over the 15 years post‐injury based on both self (*p* = .010) and other‐reported (*p* = .003) data. Across TBI severity groups, 61% (self‐report) and 66% (other report) of participants received at least one therapy by 15 years post‐injury. As expected, the severe TBI group received a significantly higher number of therapies compared to the mild (*p* = .008; *p* = .002) and moderate (*p* = .013; *p* = .035) TBI groups based on both self and other‐reported data, respectively, and in the moderate (*p* = .031) compared to the mild TBI group (other‐reported). Hence, age at injury and self‐reported number of therapies received by T5 were controlled for in subsequent analyses involving the TBI severity groups.

### Aim 1: Favourable versus less favourable psychosocial outcomes in the TBI versus TDC groups

**FIGURE 1:**
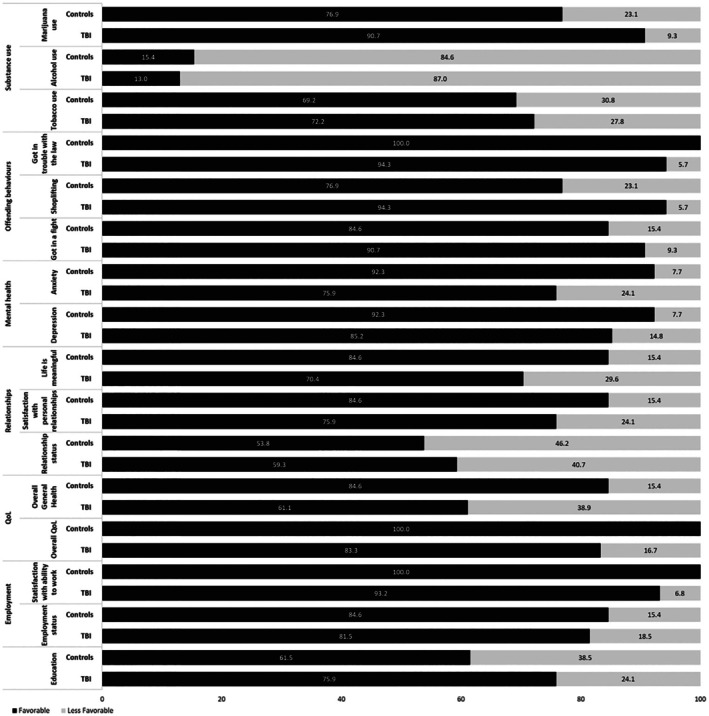
TBI and controls—differences in proportion (%) of participants with favourable and less favourable psychosocial outcomes. Favourable vs. less favourable outcomes were categorised as follows, respectively: *Education* (Undergraduate/technical/postgraduate vs. Highschool/Lower), *employment status* (employed vs. unemployed), *satisfaction with ability to work* (satisfied vs. dissatisfied), *Overall QoL* (Good vs. Poor), *Overall general health* (satisfied vs. dissatisfied), *relationships status* (in a relationship vs. not in a relationship), *satisfaction with personal relationships* (satisfied vs. dissatisfied), *life is meaningful* (Great/extremely meaningful vs. not/small/moderately meaningful), *depression/anxiety* (normal/mild vs. moderate to extremely severe), *offending behaviours* (Never vs. One or more times), *substance use* (Less often used—Never/Once/twice a month/Few times a year; Often used—At least once a week/almost every day). QoL, quality of life. Outcome areas (e.g., education) were classified as having ‘predominantly favourable outcomes’ when ≥80% of participants for a particular group (e.g., TBI) have positive outcomes and ‘predominantly less favourable outcomes’ when ≥20% of participants reported difficulties or unmet needs.

Figure 1 shows that of the 16 outcomes assessed, 50% were classified as ‘predominantly less favourable outcomes’ (i.e., ≥20% of participants reported difficulties or unmet needs) in the TBI group. Impacted areas included alcohol use, tobacco use, anxiety, all three relationship outcomes, general health, and education, with the highest rates observed for alcohol use (87.0%), relationship status (40.7%), and general health (38.9%). The TBI group showed ‘predominantly favourable outcomes’ (≥80% reported positive outcomes) in the remaining domains, with the highest rates of positive outcomes observed for non‐use of marijuana (90.7%), absence of offending behaviours (90.7%–94.3%), and satisfaction with work (93.2%).

The control group also showed ‘predominantly less favourable outcomes’ in 6 of 16 domains (37.5%): marijuana use, alcohol use, tobacco use, shoplifting, relationship status, and education.

Chi‐squared tests showed a significant group difference only for overall QoL, with more participants in the TBI group (16.7%) reporting poorer QoL compared to the control group (0%), [*χ* ^2^ = 4.207 (df = 1, *N* = 67), *p* = .040].

### Aim 2: Favourable versus less favourable psychosocial outcomes across the TBI severity groups

**FIGURE 2:**
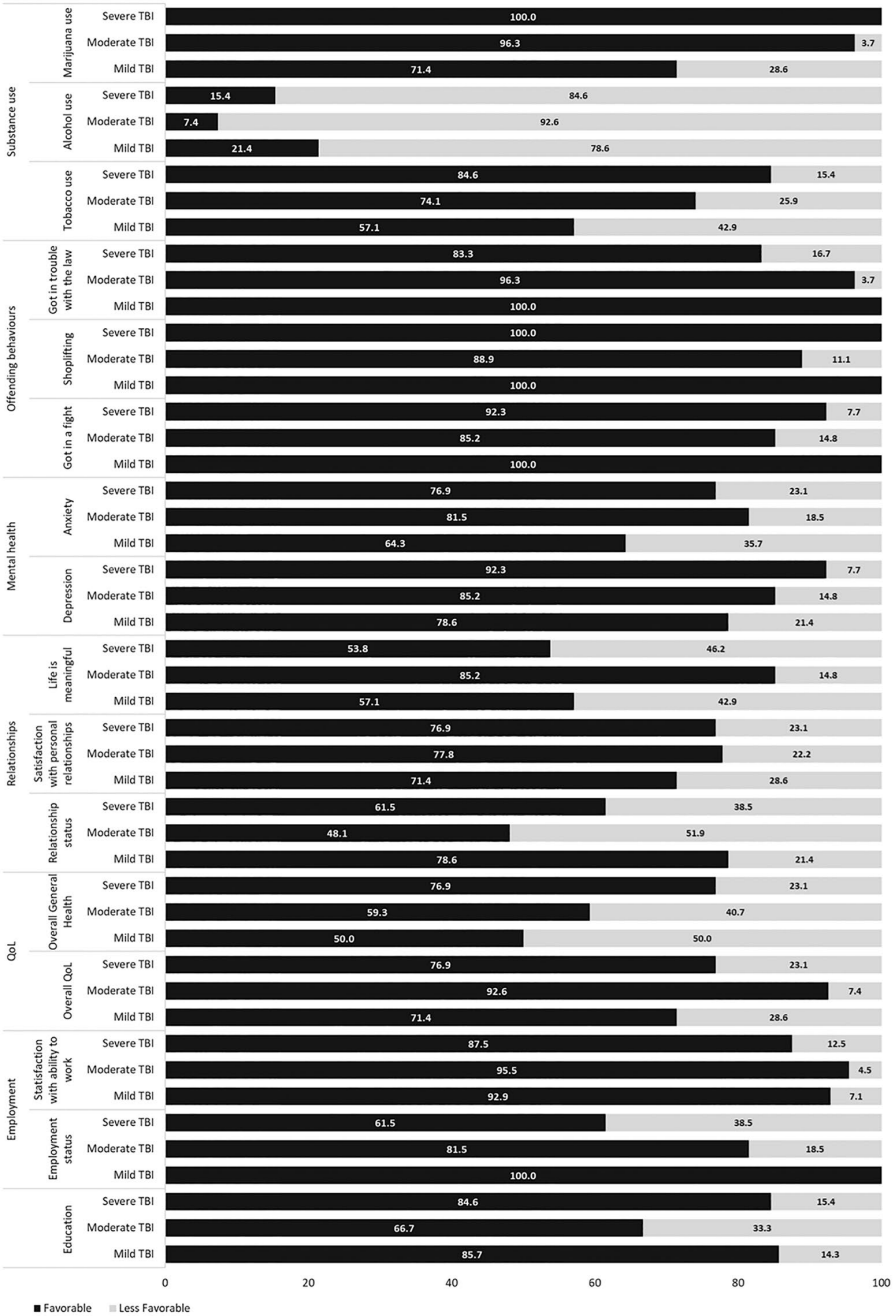
TBI severity groups—differences in proportion (%) of participants with favourable and less favourable psychosocial outcomes. Favourable vs. less favourable outcomes were categoried as follows, respectively: *Education* (Undergraduate/technical/postgraduate vs. Highschool/Lower), *employment status* (employed vs. unemployed), *satisfaction with ability to work* (satisfied vs. dissatisfied), *Overall QoL* (Good vs. Poor), *Overall general health* (satisfied vs. dissatisfied), *relationships status* (in a relationship vs. not in a relationship), *satisfaction with personal relationships* (satisfied vs. dissatisfied), *life is meaningful* (Great/extremely meaningful vs. not/small/moderately meaningful), *depression/anxiety* (normal/mild vs. moderate to extremely severe), *offending behaviours* (Never vs. One or more times), *substance use* (Less often used—Never/Once/twice a month/Few times a year; Often used—At least once a week/almost every day). QoL, quality of life. Outcome areas (e.g., education) were classified as having ‘predominantly favourable outcomes’ when ≥80% of for a group (e.g., mild TBI) have positive outcomes and ‘predominantly less favourable outcomes’ when ≥20% of participants for a group reported difficulties or unmet needs.

Analyses examining whether rates of favourable versus less favourable outcomes varied by TBI severity are presented in Figure 2. Across TBI severity groups, 12 of 16 outcome domains (75%) were classified as predominantly less favourable, including marijuana, alcohol and tobacco use, anxiety, depression, life meaning, satisfaction with personal relationships, relationship status, general health, overall QoL, employment, and education. The highest proportions of less favourable outcomes were observed for alcohol use (84.6%–92.6%), life meaning (42.9%–46.2%), relationship status (38.5%–51.9%), and general health (23.1%–50%).

By severity, the mild TBI group had the highest number of predominantly less favourable outcomes (10/16 domains; 62.5%), followed by the severe TBI group (8/16 domains—50%) and the moderate group (6/16 domains—37.5%).

Statistically significant differences emerged only for employment status and marijuana use. Follow‐up analyses showed that marijuana use was significantly higher in the mild TBI (28.6%) compared to the moderate (3.7%) and severe (0%) TBI groups [*χ* ^2^ = 8.012 (df = 2, *N* = 54), *p* = .018]. Employment also differed significantly, with unemployment rates being significantly lower in the mild group (0%) compared to the severe group (38.5%) [*χ* ^2^ = 6.608 (df = 2, *N* = 54), *p* = .014].

### Aim 3: Factors associated with psychosocial outcomes in young adults with childhood TBI

Results from Generalised Linear Models evaluating factors associated with each psychosocial outcome domain are presented in Table 3. These psychosocial domains were not significantly associated with any factor: education, QoL, mental health, or offending behaviours (all *p* > .05). Where significant relationships emerged, adjusted models were run, controlling for age at injury and self‐reported number of therapies since these variables differed significantly between the TBI severity groups.

**TABLE 3:** Factors associated with psychosocial outcomes in young adults with childhood TBI (*n* = 54).

Correlates	Education				Employment							
	Level of education				Employment status				Satisfaction with ability to work			
	*B*	SE	CI (95%)	*p*	*B*	SE	CI (95%)	*p*	*B*	SE	CI (95%)	*p*
TBI severity				0.296				0.135				0.766
Mild	−.09	1.08	−2.2, 2.0		2.12	1.21	−.2, 4.5		.31	.76	−1.2, 1.8	
Moderate	1.01	.87	−.7, 2.7		.60	.45	−.3, 1.5		.54	.73	−.9, 2.0	
Severe	‐	‐	‐		‐	‐	‐		‐	‐	‐	
Sex (male)	.20	.64	−1.0, 1.5	.749	.28	.40	−.5, 1.1	.484	−1.29	1.00	−3.2, .7	.198
Age at injury	−.17	.11	−.4, .1	.128	−.03	1.09	−.2, .2	.819	−.01	.19	−.4, .4	.975
SES at t1	.56	.34	−.1, 1.2	.101	−.86	.39	−1.6, −.1	.**024**	−.02	.66	−1.3, 1.3	.980
Family functioning at t1	.03	.05	−.1, .1	.636	.01	.05	−.1, .1	.885	.00	.07	−.1, .1	.989
FSIQ at t1	.01	.02	−.0, .1	.760	.01	.02	−.0, .1	.655	.01	.04	−.1, .1	.746
EF at t10	−.01	.03	−.1, .0	.729	−.02	.04	−.1, .01	.693	−.07	.05	−.2, .0	.138
Social skills at 10 years post‐injury	−.02	.02	−.1, .0	.397	.00	.03	−.1, .1	.932	.02	.03	−.0, .1	.596
Adaptive behaviour at t1	.00	.02	−.0, .0	.915	.02	.02	−.0, .1	.430	.04	.04	−.0, .1	.370
Therapy completed (self)	−.25	.30	−.8, .3	.411	−.38	.22	−.8, .0	.078	−.68	.41	−1.5, .1	.095
Therapy completed (other)	−.01	.22	−.4, .4	.975	−.39	.21	−.8, .0	.071	−.22	.34	−.9, .5	.526

Correlates	Quality of life								Relationships			
	Overall QoL				Overall general health				Relationship status			
	*B*	SE	CI (95%)	*p*	*B*	SE	CI (95%)	*p*	*B*	SE	CI (95%)	*p*
TBI severity				.186				.342				.342
Mild	.17	.52	−1.2, .9		−.74	.51	−1.7, .3		.50	.52	−.5, 1.5	
Moderate	.71	.53	−.3, 1.7		−.50	.45	−1.4, .4		−.34	.43	−1.2, .5	
Severe	‐	‐	‐		‐	‐	‐		‐	‐	‐	
Sex (male)	−.46	.42	−1.3, .4	.275	.10	.35	−.6, .8	.780	‐	‐	‐	‐
Age at injury	.19	.13	−.1, .5	.143	.08	.09	−.1, .3	.387	.21	.10	.0, .4	.**035**
SES at t1	−.37	.37	−1.1, .4	.319	−.20	.27	−.7, .3	.469	.02	.27	−.5, .6	.953
Family functioning at t1	−.09	.09	−.3, .1	.322	.07	.05	−.0, .2	.160	−.03	.04	−.1, .1	.543
FSIQ at t1	.00	.23	−.0, .1	.901	−.00	.02	−.0, .0	.923	−.01	.02	−.0, .0	.629
EF at t1	−.02	.03	−.1, .0	.458	.03	.03	−.0, .1	.256	.01	.02	−.0, .1	.700
Social skills at 10 years post‐injury	.00	.02	−.0, .1	.927	−.00	.02	−.0, .0	.954	.04	.03	−.0, .1	.096
Adaptive behaviour at t1	.04	.03	−.0, .1	.162	−.01	.02	−.0, .0	.700	−.01	.02	−.1, .0	.658
Therapy completed (self)	−.14	−.25	−.6, .4	.584	.24	.21	−.2, .7	.253	.40	.23	−.1, .9	.083
Therapy completed (other)	.17	.30	−.4, .8	.574	.14	.18	−.2, .5	.420	.02	.17	−.3, .4	.886

Correlates	Relationships								Mental health			
	Satisfaction with personal relationships				Life is meaningful				Anxiety			
	*B*	SE	CI (95%)	*p*	*B*	SE	CI (95%)	*p*	*B*	SE	CI (95%)	*p*
TBI severity				0.901				0.060				0.486
Mild	−.17	.52	−1.2, .9		.08	.48	−.9, 1.0		−.37	.52	−1.4, .6	
Moderate	.03	.47	−.9, .9		.95	.46	.1, 1.8		.16	.48	−.8, 1.1	
Severe	‐	‐	‐		‐	‐	‐		‐	‐	‐	
Sex (male)	−.12	.37	−.9, .6	.750	−.22	.36	−.9, .5	.551	−.61	.39	−1.4, .1	.113
Age at injury	.19	.11	−.0, .4	.098	.21	.11	−.0, .4	.052	−.01	.10	−.2, .2	.930
SES at t1	−.15	.32	−.8, .5	.626	−.19	.30	−.8, .4	.522	−.48	.31	−1.1, .1	.126
Family functioning at t1	−.01	.05	−.1, .1	.766	.03	.04	−.0, .1	.427	−.03	.05	−.1, .1	.525
FSIQ at t1	−.02	.02	−.1, .0	.317	−.00	.02	−.0, .0	.870	.01	.02	−.0, .0	.738
EF at t1	−.03	.03	−.1, .0	.251	−.05	.03	−.1, .0	.101	.03	.03	−.0, .1	.257
Social skills at 10 years post‐injury	.02	.02	−.0, .1	.344	−.01	.02	−.1, .0	.489	.01	.02	−.0, .0	.796
Adaptive behaviour at t1	.02	.02	−.0, .1	.442	.01	.02	−.0, .1	.540	−.02	.02	−.0, .1	.233
Therapy completed (self)	−.03	.21	−.4, .4	.872	−.33	.20	−.7, .1	.105	.06	.22	−.4, .5	.788
Therapy completed (other)	−.08	.20	−.5, .3	.676	−.01	.20	−.4, .4	.977	‐	‐	‐	‐

Correlates	Mental health				Offending behaviours							
	Depression				Got in a fight				Shoplifting			
	*B*	SE	CI (95%)	*p*	*B*	SE	CI (95%)	*p*	*B*	SE	CI (95%)	*p*
TBI severity				.606				.456				.422
Mild	−.63	.64	−1.9, .6		.98	1.26	−1.5, 3.5		‐	1.70	−3.3, 3.3	
Moderate	−.38	.59	−1.5, .8		−.38	.59	−1.5, .8		−1.19	1.29	−3.7, 1.3	
Severe	.25	.48	−.7, 1.2		‐	‐	‐		‐	‐	‐	
Sex (male)	−.33	.42	−1.2, .5	.444	−.73	.54	−1.8, .3	.172	−.35	−.57	−1.5, .8	.543
Age at injury	.00	.12	−.2, .2	.979	−.07	.14	−.3, .2	.650	.13	.19	−.2, .5	.487
SES at t1	−.73	.39	−1.5, .0	.057	−.70	.47	−1.6, .2	.137	.31	.60	−.9, 1.5	.600
Family functioning at t1	−.02	.05	−.1, .1	.705	−.06	.09	−.2, .1	.511	−.04	.13	−.3, .2	.781
FSIQ at t1	.02	.02	−.0, .1	.454	−.00	.03	−.1, .1	.899	.02	.03	−.0, .1	.473
EF at t1	.04	−.03	−.0, .1	.257	.04	.03	−.0, .1	.206	−.07	.06	−.2, .0	.192
Social skills at 10 years post‐injury	.02	.02	−.0, .1	.331	.02	.03	−.0, .1	.413	.02	.04	−.1, .1	.501
Adaptive behaviour at t1	.00	.02	−.0, .0	.918	.02	.03	−.0, .1	.586	−.01	.04	−.1, .1	.893
Therapy completed (self)	.07	.28	−.5, .6	.812	−.13	.27	−.7, .4	.643	.45	.60	−.7, 1.6	.459
Therapy completed (other)	.03	.27	−.5, .5	.916	−.15	.24	−.6, .3	.525	.02	.31	−.6, .6	.938

Correlates	Offending behaviours				Substance use							
	Got in trouble with the law				Tobacco use				Alcohol use			
	*B*	SE	CI (95%)	*p*	*B*	SE	CI (95%)	*p*	*B*	SE	CI (95%)	*p*
TBI severity				0.290				0.923				0.235
Mild	1.44	1.23	−1.0, 3.9		−.22	.82	−1.8, 1.4		−1.39	.82	−3.0, .2	
Moderate	.81	.62	−.4, 2.0		.54	.73	−1.4, 1.5		−.73	.71	−2.1, .7	
Severe	‐	‐	‐		‐	‐	‐		‐	‐	‐	
Sex (male)	−.35	.57	−1.5, .8	.543	.17	.59	−1.0, 1.3	.771	−.90	.56	−2.0, .2	.106
Age at injury	−.10	.17	−.4, .2	.566	−.04	.09	−.2, .1	.697	−.28	.10	−.5, −.1	.**006**
SES at t1	.09	.58	−1.0, 1.2	.873	−.04	.28	−.6, .5	.897	.65	.31	.04, 1.3	.**035**
Family functioning at t1	−.03	.11	−.2, .2	.750	−.01	.04	−.1, .1	.849	−.03	.04	−.1, .1	.442
FSIQ at t1	.04	.03	−.0, .1	.172	−.02	.02	−.0, .1	.423	−.00	.02	−.0, .0	.872
EF at t1	−.07	.06	−.2, .0	.192	−.03	.03	−.1, .0	.384	−.00	.03	−.1, .1	.942
Social skills at 10 years post‐injury	.02	.04	−.1, .1	.501	−.01	.03	−.1, .0	.752	−.04	.03	−.1, .0	.180
Adaptive behaviour at t1	.03	.04	−.0, .1	.433	−.01	.02	−.0, .0	.717	.02	.02	−.0, .1	.347
Therapy completed (self)	‐	‐	‐	‐	.18	.23	−.3, .6	.432	.07	.19	−.3, .4	.723
Therapy completed (other)	−.21	.30	−.8, .4	.488	.03	.17	−.3, .0	.845	−.07	.17	−.4, .3	.669

Correlates	Substance use			
	Marijuana use			
	*B*	SE	CI (95%)	*p*
TBI severity				‐
Mild	‐	‐	‐	
Moderate	‐	‐	‐	
Severe	‐	‐	‐	
Sex (male)	−.48	1.13	−2.7, 1.7	.674
Age at injury	−.23	.17	−.6, .1	.197
SES at t1	.45	.54	−.6, 1.5	.409
Family functioning at t1	.01	.06	−.1, .1	.877
FSIQ at t1	.00	.03	−.1, .1	.968
EF at t1	.01	.05	−.1, .1	.855
Social skills at 10 years post‐injury	−.01	.05	−.1, .1	.819
Adaptive behaviour at t1	.01	.03	−.1, .1	.702
Therapy completed (self)	.29	.42	−.5, 1.1	.493
Therapy completed (other)	.21	.36	−.5, .9	.552

*Note*: Bold values represent statistically significant differences (*p* < 0.05).

Abbreviations: Adaptive behaviour, adaptive behaviour composite (Vineland); Social skills at 10 years post‐injury, Behaviour Assessment System for Children (BASC); EF, executive functioning, behaviour rating inventory of executive function (BRIEF); FSIQ, full scale intelligence quotient; SES, socio‐economic status.

Statistically significant predictors were identified for employment status, relationships, and alcohol use. Lower SES at the time of injury was significantly associated with a higher likelihood of being employed, compared to higher SES at the time of injury in both unadjusted and (*β* = −.86, Wald *χ* ^2^ = 5.09, *p* = .024) adjusted models (*β* = −.90, Wald *χ* ^2^ = 4.20, *p* = .040). Being in a romantic relationship was significantly associated with younger age at injury (*β* = −.21, Wald *χ* ^2^ = 4.42, *p* = .035), but this relationship was no longer statistically significant after controlling for self‐reported number of therapies (*β* = .22, Wald *χ* ^2^ = 3.09, *p* = .079). More frequent alcohol use was significantly associated with younger age at injury (*β* = −.28, Wald *χ* ^2^ = 7.56, *p* = .006) and lower SES at the time of injury (*β* = .65, Wald *χ* ^2^ = 4.42, *p* = .035), and the results remained significant in the adjusted models (*β* = −.53, Wald *χ* ^2^ = 8.64, *p* = .003), (*β* = .78, Wald *χ* ^2^ = 4.06, *p* = .044), respectively.

## DISCUSSION

This study aimed to identify the proportion of young adults with childhood TBI who report favourable and less favourable psychosocial outcomes, relative to a TDC group, and to explore factors associated with these outcomes at 20 years following childhood TBI. Across the 16 outcome domains assessed, 24%–87% of the TBI group showed vulnerabilities in 75% of areas—substantially higher than the 37.5% impacted in the typically developing control (TDC) group. Although the specific domains varied by TBI severity, the mild TBI group showed the greatest vulnerability (62.5% of domains impacted; 21%–78.6% of participants), followed by the severe group (50% of domains impacted). More frequent alcohol use was associated with younger age at injury and lower SES, while individuals from lower SES backgrounds were more likely to be employed.

These findings indicate that while some young adults with childhood TBI achieve favourable psychosocial outcomes, a substantial proportion (up to 90% in certain domains) require ongoing support to manage long‐term challenges, particularly when injury occurs at a younger age.

### Favourable psychosocial outcome areas and associated factors

Four outcomes (25%) were classified as predominantly favourable, with 85%–100% of TBI participants showing positive outcomes: all three offending behaviour outcomes and satisfaction with ability to work. This highlights a nuanced picture in which some individuals with childhood TBI demonstrate strengths and achieve positive outcomes in specific domains, despite difficulties in other areas.

Approximately 88%–96% of young adults with childhood TBI were satisfied with their ability to perform their work duties, demonstrating confidence in their abilities and skills, which is similar to the 100% reported in the TDC group. Less than 17% of participants with TBI engaged in offending behaviours, including fighting (0%–14.8%), shoplifting (0%–11.1%), and trouble with the law (0%–16.7%). These rates are similar to the TDC group (0–15.4%), but significantly lower than previously reported in other clinical and community samples with childhood TBI (20%–69%), which often show greater risk of offending compared to controls (Bellesi et al., 2019; Fishbein et al., 2016; McKinlay et al., 2014). A possible explanation for this disparity is that most previous studies focused on people in the criminal justice system (Bellesi et al., 2019; Hughes et al., 2015). Alternatively, the younger average age at injury in our cohort (<10 years) may be protective, consistent with findings from a large Swedish study (*n* = 22,914) showing lower risk of offending in individuals injured before age 16 compared to those injured later (Fazel et al., 2011).

### Less favourable psychosocial outcomes areas and associated factors

Findings supported hypothesis one, with the TBI group reporting predominantly less favourable outcomes (i.e., ≥20% of participants reported difficulties or unmet needs) in 75% of psychosocial domains, affecting 24%–87% TBI participants. Although higher than the 37.5% of impacted domains in the TDC group, only overall QoL differed significantly, with greater vulnerability identified in the TBI group. In contrast to hypothesis two, the anticipated dose–response relationship was not supported. The mild TBI group showed greater vulnerability across multiple domains (62.5% domains), followed by the severe TBI group (50% domains), with significant differences limited to marijuana use (higher in mild TBI) and unemployment (higher in severe TBI). While further research is needed to examine contributing factors, these outcomes in the mild TBI group may reflect limited access to support and therapy compared with the more severe groups, consistent with evidence that untreated mild TBI can lead to persistent long‐term difficulties (Emery et al., 2016; Lumba‐Brown et al., 2018). Across the TBI group, 12 areas of concern were identified: education, employment, general health, overall QoL, alcohol, tobacco, and marijuana use, anxiety, depression, life meaning, satisfaction with personal relationships, and relationship status. We discuss these findings in the paragraphs that follow.

Approximately 24%–33%% of our TBI sample, particularly those with moderate TBI experienced less favourable educational outcomes, and completed only high school (i.e., Year 12 or lower), while the remainder attained education beyond high school. Although this rate was lower than reported in the TDC group (38.5%), the proportion of individuals attaining up to high school education is notably higher than the 15.5% reported in Australian adults (25–64 years) as of 2021 (Australian Institute of Health and Welfare, 2023). In a prior Australian study, 36.6% of young adults with childhood TBI completed only high school education (Anderson et al., 2010), and other previous studies have reported lower educational attainment in individuals who sustained TBI in childhood (Catroppa et al., 2009; Crowe et al., 2012; Durber et al., 2017; Ilie et al., 2020). These findings suggest that a subgroup of young people with childhood TBI may be at greater risk of lower educational attainment, highlighting the importance of early academic supports to improve long‐term outcomes since higher education (e.g., diploma or degree) was recently linked with better employment prospects among adults in Australia (Lumba‐Brown et al., 2018).

Unemployment was reported in 38.5% of the TBI group, with the severe TBI group being the only group affected, highlighting a need for greater vocational supports for young people with severe TBI who are able to work. This rate is much higher than the 15% reported in the TDC group, the 9% youth unemployment rate reported in Australia (Jobs and Skills Australia, 2025), and contradicts findings from a fairly recent study reporting a 12.3% unemployment rate in individuals who sustained TBI in childhood (Visnick et al., 2023). The finding, however, aligns with studies reporting higher unemployment rates following childhood TBI (30%–49%) (Anderson et al., 2009, 2010), and in those with severe TBI (Anderson et al., 2009). Interestingly, lower SES at the time of injury was associated with a greater likelihood of being employed (Aliaga et al., 2024). Compared to those from high SES families, those from low SES families may have fewer supports (e.g., financially), leading the latter to seek jobs out of economic necessity. It is also possible that those from high SES families were pursuing further education/other interests rather than working at the time of this study. More research is needed to understand the relationship between SES and employment outcomes in the very long term after childhood TBI.

A substantial proportion of the TBI participants (23%–50%) reported less favourable outcomes in the QoL domain, affecting general health and overall QoL. Poor general health and QoL were reported in 23%–50% of the TBI sample, which is much higher than the 0%–15.4% reported in the control group or the 8.1%–8.5% reported in similar age groups (15–34 years) in Australia (Australian Bureau of Statistics, 2018). The low QoL reported in this sample, however, aligns with several previous childhood TBI studies (Anderson et al., 2009, 2010; Rosema et al., 2014).

Another 21%–36% of young adults with childhood TBI experienced concerns in the mental health domain, particularly including anxiety and depression symptoms. Lower rates were reported in the TDC group (7.7%–15.4%) and in 16–34‐year‐olds in Australia (7.5%–24.4%) (Australian Bureau of Statistics, 2022a), highlighting greater risk of mental challenges in the very long‐term following childhood TBI. These findings suggest a need for tailored supports to address the unmet mental health needs of this group.

Substance use was a particular concern in the TBI group, with high proportions of participants using these substances more than once a week or almost daily: alcohol (79%–93%), tobacco (26%–43%), and marijuana (29% in the mild TBI group). These rates are comparatively higher than those reported in Australian young adults (18–39 years) who frequently use alcohol (13.8%–14.7%) (Australian Bureau of Statistics, 2022b) or tobacco (6.0%–8.9%) (Greenhalgh et al., 2024) as of 2022–2023. This is concerning given the known negative impacts of substance use on behaviour and mental health following paediatric TBI (Bellesi et al., 2019; Ilie et al., 2015; Kennedy et al., 2017). The findings highlight the need to explore factors influencing these behaviours besides low SES (at T1) and younger age at injury, which were implicated in this study. Further research into these implicated factors and the role of peer influence and transition‐related heightened responsibilities/expectations could offer further insights into how these behaviours are encouraged and sustained in people who experienced TBI in childhood, informing potential intervention pathways.

The relationship domain emerged as a key concern in the TBI group: over 40% of participants felt life was not meaningful, over 20% were dissatisfied with personal relationships, and 20%–52% were not in a relationship/did not have a partner. Notably, a high rate of those with moderate and severe TBI (39%–52%) did not have a partner, highlighting the potential role of cognitive, behavioural, and social difficulties associated with such severe injuries. While further research is needed to explore these findings, the results may be related to several interconnected factors, including TBI‐induced cognitive, language, mental health, emotional regulation, and behaviour problems (Jones et al., 2021; Rosema et al., 2012; Ryan, Noone, et al., 2019), which can affect relationships through their impacts on social function (e.g., low self‐esteem and social isolation) and communication (Anderson et al., 2013; Atay et al., 2016).

Young adults who sustained TBI later in childhood were less likely to be in a relationship. This contrasts findings from a similar study showing this trend in those who experienced TBI at a younger age (Anderson et al., 2010), and may be attributed to communication, language, and self‐esteem/self‐worth deficits associated with childhood TBI (Anderson et al., 2013; Atay et al., 2016). Low self‐esteem has been reported in individuals who sustained TBI in childhood as early as 12 months post‐injury, which was linked to poorer friendship quality and greater peer‐related loneliness (Khan et al., 2023). It is therefore possible that individuals in the older injury group have lower self‐esteem due to a more vivid recollection of their injury and recovery experiences (Anderson et al., 2013; Atay et al., 2016), which could significantly impact their relationship outcomes. The fact that this relationship was no longer significant after controlling for the number of self‐reported therapies received by 15 years post‐injury highlights the need for ongoing therapy support for these young people.

### Limitations

A few study limitations need to be taken into account when interpreting these findings. The high attrition rate in this 20‐year follow‐up (only 31% of the original TBI sample participated) coupled with the underrepresentation of males and people from high SES backgrounds in the 20‐year participate group limits the generalizability of the results to all young adults with childhood TBI. While the attrition rate is relatively high, it aligns with rates reported in longitudinal paediatric TBI studies, which commonly range between 20% and 60%; with extended studies like this one often having higher rates (Blaha et al., 2015). The small sample size limits generalizability of the findings, and the higher participation of low SES at the time of injury compared to high SES members of this cohort in this follow‐up also poses a potential risk of bias. We also did not have the scope to control for some factors known to influence psychosocial outcomes in the long‐term after TBI, such as maternal factors (marital status, maternal age, maternal smoking) (Sariaslan et al., 2016; Visnick et al., 2023). Single/multiple items were used to evaluate most of the outcomes in this study, which limits the generalizability and robustness of the findings. Given concerns about limited insight, especially following more severe TBI, our results may have been impacted by this factor since outcomes were self‐reported. In the absence of a definitive prevalence cut‐off for defining less favourable outcome rates in childhood TBI, an established rate (≥20%) from the obesity literature was used (Lobstein & Jewell, 2022), but using a rate defined for the TBI literature would be ideal. Future studies should aim to establish a prevalence threshold specific to TBI outcomes to improve the robustness of such classifications.

### Implications for research and clinical practice

Over the years, several studies have reported poor psychosocial outcomes after childhood TBI, but the nature of these outcomes in the very‐long term post‐injury are poorly characterised, highlighting the need for similar studies. Periodic screening is also needed to identify those at risk of poor outcomes in these compromised domains and to develop/provide evidence‐based interventions to support these vulnerable groups. While functional outcomes (e.g., adaptive functioning) typically predict outcomes after childhood TBI (Anderson et al., 2012; Catroppa et al., 2008; Ryan et al., 2015), they did not do so in this young adult group, highlighting the need for further research on these relationships in adulthood. The poor relationship outcomes reported in the current cohort highlight the need for ongoing monitoring of emerging language, social, and communication challenges and support throughout school years and into adulthood following childhood TBI. Similar approaches are needed to support young people who sustained TBI in childhood with regard to improving their general health (HRQoL), anxiety symptoms, and substance use.

## CONCLUSIONS

Conclusions about the long‐term impact of childhood TBI can only be drawn in adulthood, and this paper contributes to that body of literature by highlighting the lasting effects of childhood TBI on psychosocial outcomes. At two decades post‐injury, the findings indicate that while most young adults with childhood TBI have favourable outcomes in some psychosocial domains (e.g., offending behaviour), a relatively large proportion (24%–87%, depending on the outcome area) experience concerns in domains such as relationships, general health, anxiety, and substance use, influenced by factors such as younger age at injury. These findings add to the growing evidence challenging the previously held belief that children “catch up” after TBI and that sustaining TBI at a younger age is associated with better recovery. The results emphasize the need for ongoing monitoring and care for individuals who sustain childhood TBI, as well as early identification of those at risk of poor outcomes in affected domains (e.g., education and relationships). Developing and providing evidence‐based interventions is crucial to mitigate the risk of less favourable psychosocial outcomes in the very long‐term post‐injury.

## TRANSPARENCY, RIGOUR, AND REPRODUCIBILITY SUMMARY

To increase participation in this 20‐year follow‐up, extensive efforts were made to invite all original participants in this cohort (*n* = 172), including using most recent contact details from their voting/electoral profiles (using information officially provided by the Victorian Electoral Commission, Australia). A detailed account of reasons for non‐participation is provided in the Methods section and Table S2 for transparency. Basic demographic details of original participants who did not participate in this 20‐year follow‐up have also been provided in Table S1. Considering the long‐term nature of this follow‐up (i.e., 20 years), the study was not formally registered, and power calculations were not performed for the current analyses. We included all valid data from participants who were available for this follow‐up. Each participant in this study received a study completion letter, including overall outcomes across the group (group data), as well as their unique individual outcome, and no identifiable information was included in the group data. Missing data points have been noted in the footnotes of Table 2. Confidence intervals and *p*‐values have been reported in Table 3 to provide statistical significance.

## AUTHOR CONTRIBUTIONS

**Edith Botchway‐Commey:** Conceptualization; investigation; writing – original draft; methodology; visualization; software; formal analysis; project administration; data curation; validation. **Nicholas P. Ryan:** Conceptualization; investigation; writing – review and editing. **Louise Crowe:** Conceptualization; writing – review and editing; investigation. **Celia Godfrey:** Conceptualization; investigation; funding acquisition; writing – review and editing; methodology; supervision; data curation. **Harry Macleod:** Writing – review and editing. **Stephen Hearps:** Writing – review and editing; formal analysis. **Vicki A. Anderson:** Supervision; writing – review and editing; methodology; investigation; conceptualization; funding acquisition. **Cathy Catroppa:** Investigation; conceptualization; funding acquisition; methodology; writing – review and editing; supervision; data curation.

## FUNDING INFORMATION

This project is supported by the Australian National Health and Medical Research Council project and fellowship funding, Victorian Operational Infrastructure fund.

## CONFLICT OF INTEREST STATEMENT

The authors declare they have no competing interests.

## Supporting information


Table S1.

Table S2.

## Data Availability

The data used in this paper are not publicly available but can be made available following ethics approval from the RCH Human Research Ethics Committee.
