# Supplementary material for: Psychosocial outcomes in young adults with childhood traumatic brain injury: A 20‐year follow‐up study
Source: J Neuropsychol. 2026 Apr 15;20(2):445–67. doi: 10.1111/jnp.70042 (PMC13250358; doi:10.1111/jnp.70042)
Supplement: Supplementary file 1 — Table S1. [file JNP-20-445-s001.docx]

#### **Supplemental Table 1**. Characteristics Of Participants Seen, And Not Seen At 20-Year Follow-Up

|  | *TBI* | | |  | *TDC* | | |
| --- | --- | --- | --- | --- | --- | --- | --- |
| *N* | Seen at 20 years  54 | Not seen at 20 years  118 | *P* |  | Seen at 20 years  13 | Not seen at 20 years  22 | *P* |
| Male, *n (*%) | 27 (50) | 87 (73.7) | **.002** |  | 8 (61.5) | 10 (45.5) | .358 |
| Age at injury, mean *(SD)* | 6.46 (3.3) | 7.5 (3.4) | .061^a^ |  | - | - | - |
| Age at follow-up, mean *(SD)* | 27.6(3.3) | 28.2 (3.5) | .238^a^ |  | 25.88 (2.2) | 25.92 (2.2) | .960^b^ |
| Severity group  *Mild*, *n* (%)  *Moderate*, *n* (%)  *Severe*, *n* (%) | 14 (25.9)  27 (50)  13 (24.1) | 30 (25.4)  54 (45.8)  34 (28.8) | .799 |  | - | - | - |
| SES (t1), mean (SD) | 4.12 (1.0) | 4.42 (.8) | **.032^a^** |  | 3.44 (1.2) | 3.9 (.7) | .159^a^ |
| Cause of injury  *MVA: Motor vehicle*, *n* (%)  *MVA: Bicycle*, *n* (%)  *MVA: Pedestrian*, *n* (%)  *Fall*, *n* (%)  *Head hits object*, *n* (%)  *Violence*, *n* (%)  *Machinery*, *n* (%) | 9 (16.7)  10 (18.5)  7 (13)  23 (42.6)  3 (5.6)  1 (.8)  0 | 16 (13.6)  17 (14.4)  33 (28)  36 (30.5)  15 (12.7)  1 (1.9)  1 (1.9) | .119 |  | - | - | - |
|  |  |  |  |  |  |  |  |
| Adaptive functioning (t1) | 111.2 (.15.7) | 107.5 (15.9) | .186 |  | - | - | - |
| FSIQ (t1) | 103.8 (16.1) | 93.9 (16.1) | **<.001** |  | - | - | - |

Results are based on Chi-squared test statistic unless otherwise specified. Boldface represents significant group companions (*p* < 0.05).

^a^Mann-Whitney *U* test results.

^b^Independent sample *t*-test results.

FSIQ, full-scale IQ; MVA, motor vehicle accident; SES (t1), Socio-economic status at time 1 (missing values for 45 participants replaced with participants’ group mean); TBI, traumatic brain injury.
