# Supplementary material for: Psychosocial outcomes in young adults with childhood traumatic brain injury: A 20‐year follow‐up study
Source: J Neuropsychol. 2026 Apr 15;20(2):445–67. doi: 10.1111/jnp.70042 (PMC13250358; doi:10.1111/jnp.70042)
Supplement: Supplementary file 2 — Table S2. [file JNP-20-445-s002.docx]

**Supplemental Table 2.** **Reasons for dropout/loss to follow-up**

| **Follow-up** | **Reasons for dropout/loss to follow-up** |
| --- | --- |
|  | TBI |
| *Previous follow-ups:* | Dropouts (n=9). Reasons: unwillingness to continue the study (8) and deceased (1). |
| *Current follow-up (20 years post-injury)* | Of the **166** TBI participants invited for the 20-year follow-up, **54** provided data for this study. Reasons for not participating were:  time constraints (1), loss of interest (1), deceased (2); declined participation (13); declined participation after consenting (3); could not be contacted even with updated state electoral registration information (90); did not complete some of the questionnaires presented in this study (2). |
|  | TDC |
| *Previous follow-ups:* | Dropouts (n=2). Reasons: unwillingness to continue the study (1) and deceased (1). |
| *Current follow-up (20 years post-injury)* ***- 30 participated out of 35 invited*** | Of the **30** TBI participants invited for the 20-year follow-up, **13** provided data for this study. Reasons for not participating were:  time constraints (1), loss of interest (1), deceased (2); declined participation (13); declined participation after consenting (3); could not be contacted even with updated state electoral registration information (90); did not complete some of the questionnaires presented in this study (2). |
